# High-Performance Multifunctional rPA6/rCFRP/rGraphite Hybrid Composites from Recycled Industrial Waste

**DOI:** 10.1021/acspolymersau.5c00178

**Published:** 2026-01-21

**Authors:** Erick Gabriel Ribeiro dos Anjos, Rieyssa Maria de Almeida Corrêa, Thiely Ferreira da Silva, Alan Silva dos Santos, Larissa Stieven Montagna, Juliano Marini, Luiz Antonio Pessan, Mirabel Cerqueira Rezende, Fabio Roberto Passador

**Affiliations:** † Department of Science and Technology, 28105 Federal University of São Paulo (UNIFESP), 330 Talim St., São José dos Campos, SP 12231-280, Brazil; ‡ Graduate Program in Materials Science and Engineering, 67828 Federal University of São Carlos (UFSCar), Rodovia Washington Luís, Km 235, São Carlos, SP 13565-905, Brazil; § 74360 Technological Institute of Aeronautics (ITA), Fundamental Sciences Division, Marechal do Ar Eduardo Gomes square, 50, Vila das Acácias, São José dos Campos, SP 12228-900, Brazil

**Keywords:** Recycling, Composites, Carbon fiber reinforced polymer, Graphite, Polyamide 6

## Abstract

Environmental concerns and the global shift toward a more sustainable and circular economy have increased the demand for economically viable materials derived from industrial waste. In this study, high-value engineering materials discarded from different industries were repurposed to develop new multifunctional hybrid composites. The selected postindustrial waste included polyamide 6 (rPA6) from automotive plastic washers, recycled graphite (rGra), and recycled carbon fiber reinforced polymer (rCFRP) epoxy-based composites originating from the aerospace sector. These materials were separately ground using a knife mill and subsequently compounded via extrusion. The fillers (rGra and rCFRP) were incorporated individually and as hybrids (rCFRP:rGra, 1:1 wt %) at total loadings of 5, 10, and 20 wt % in the rPA6 matrix. The composites were characterized in terms of morphology, rheology, mechanical performance (tensile test), thermal behavior (differential scanning calorimetry—DSC), electrical conductivity, and electromagnetic properties. The mechanical results revealed a notably high ultimate tensile strength of 126 MPa and an elastic modulus of 4.8 GPa for the rPA6/rCFRP (20 wt %) composition, suggesting strong interfacial adhesion promoted by secondary interactions between the epoxy resin coating on the rCFRP and the rPA6 matrix. Electrical conductivity measurements on composite films showed values from 10^–5^ to 10^–1^ S.cm^–1^ for the hybrid and rCFRP-filled compositions, indicating their potential for antistatic (anti-ESD) applications. Although rGra exhibited lower mechanical and electrical performance than rCFRP at the evaluated contents, it was less detrimental to processability, making the hybrid formulations more balanced candidates for real-world applications. Overall, this study demonstrates a promising strategy for upcycling industrial waste into high-value, multifunctional composites, thereby contributing to resource efficiency and waste minimization across various industrial sectors.

## Graphical abstract

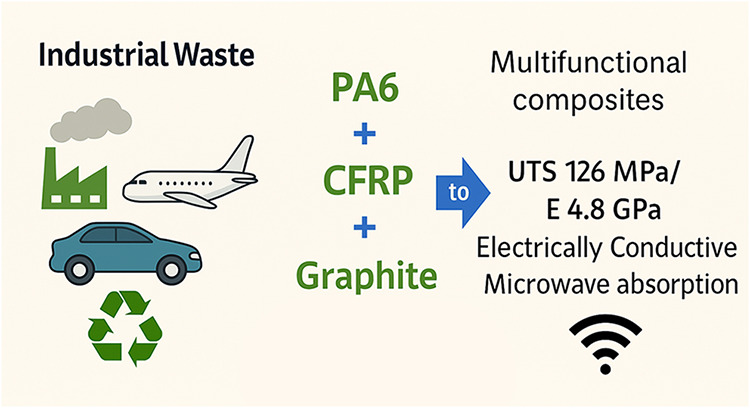

## 1 Introduction

In materials manufacturing, the principles of Reduce, Reuse, and Recycle are fundamental not only for achieving sustainability and environmental targets but also for economic viability, as they help maximize profitability and maintain competitiveness in the current market.^1,2^ Fiber-reinforced polymers (FRPs) have revolutionized modern engineering by offering high strength-to-weight ratios, elevated specific moduli, and remarkable thermal resistance. These features make FRPs an attractive alternative to metals for structural components in the aerospace, automotive, and energy sectors.^3,4^ Among these materials, carbon fiber-reinforced polymers (CFRPs) stand out for their ability to enhance fuel efficiency in transportation applications. However, CFRPs also present certain drawbacks, including anisotropic mechanical behavior, high production costs, and increasing concerns regarding waste management, both during manufacturing and at the end-of-life stages.^3^

In the aerospace industry, an alarming amount of CFRP waste is generated, with scrap rates reaching up to 40% of total prepreg materials in manufacturing processes such as hand lay-ups.^3,5^ This challenge is further complicated using thermoset matrices, particularly cured epoxy resins, which cannot be dissolved or remelted for reshaping, making recycling and reuse difficult. In such cases, the available recovery routes for these high-value materials mainly involve secondary or tertiary recycling strategies, which typically result in fiber shortening and, consequently, the downcycling of CFRP.^3,6^

The literature highlights the primary recycling methods for CFRPs, including mechanical, chemical, and thermal processes.^3,5^ Among these, mechanical recycling, which involves grinding and reusing the material as a filler, offers several advantages, including environmental compatibility, the absence of hazardous reagents or extreme process conditions, and the potential to recover both the matrix and the fiber.^3^ Moreover, it is scalable and straightforward, requiring neither specialized equipment nor highly trained labor. However, this approach typically results in a significant loss of the original mechanical properties, since the grinding process breaks down the carbon fiber, which limits its applicability in high-performance composites and confines the recycled material primarily to filler or other low-value applications.^3^ In this scenario, a creative recycling route reported in the literature involves using recycled CFRP (rCFRP) as a multifunctional filler in polymer matrices.^3,7^ This approach can enhance the mechanical, thermal, electrical, and even electromagnetic performance of the host polymer. The strategy offers multiple advantages: it minimizes waste generation and disposal, reduces energy consumption and the use of nonrenewable resources, and produces nontoxic, high-value materials with electromagnetic functionality.^8^

For example, da Silva et al.^6^ demonstrated that incorporating recycled carbon fiber (CF) from recycled composites into a polyamide 6 (PA6) matrix via extrusion significantly improved the mechanical performance. The resulting composites achieved a tensile strength of 110 MPa and an elastic modulus of 3.8 GPa with the addition of 20 wt % of the recycled CF composite, compared to 62 MPa and 2.0 GPa for neat PA6. Moreover, the material exhibited an electromagnetic attenuation of 16 dB, evidencing its multifunctional potential.^6^ Similar improvements in mechanical properties have been reported by other authors,^9–11^ further reinforcing PA6 as an attractive thermoplastic matrix for CFRP recycling.

PA6 is a polar engineering thermoplastic known for its high strength, derived from strong secondary hydrogen bonding between carboxyl and amide groups. However, this same polarity also promotes hygroscopic behavior.^12^ Fiber-reinforced PA6 is widely used in the automotive and aerospace industries for technical components requiring high strength and a broad operating temperature range.^4,9,12^ Additionally, recycled PA6 itself represents a common industrial waste stream, and its combination with recycled CFRP offers an upcycling pathway for PA6 while simultaneously downcycling CFRP, optimizing material circularity.^13^

Recently, several studies have explored strategies to further enhance the performance of ground CFRP-based composites by incorporating additional conductive fillers, such as carbon nanotubes (CNTs), to improve their electromagnetic interference shielding effectiveness (EMI SE).^6,7,14,15^ Pu et al.^7^ investigated epoxy composites containing up to 20 wt % ground CFRP and 5 wt % CNTs, achieving promising EMI SE values of 21.9 dB. Similarly, dos Santos et al.^14^ evaluated epoxy composites with 0.5–1.0 wt % CNTs and up to 30 wt % ground CFRP, reporting EMI SE values up to 25 dB, both measured within the X-band frequency range (8.2–12.4 GHz).

In this context, beyond focusing solely on CFRP as aerospace waste, graphite also represents an electrically conductive carbon-based material that is widely used in the aerospace sector, particularly graphite combined with other materials to produce brake components, due to its high thermal conductivity and intrinsic lubricating properties.^16–18^ During the machining of graphite parts, a considerable amount of scrap contaminated with machining oil is generated.^19^ Zaggo et al.^19^ addressed this issue by thermally treating graphite waste to remove residual oil, grinding it to obtain recycled graphite (rGra), and incorporating it into a poly­(trimethylene terephthalate) (PTT) matrix via extrusion at 1, 3, 5, 10, and 20 wt %. The resulting composites, designed for antistatic (ESD) packaging applications, exhibited notable improvements in mechanical properties and electrical conductivity, with a percolation threshold of 5 wt % rGra. Furthermore, graphite may also serve as an effective conductive filler to enhance the electromagnetic functionality of polymer matrices.^20^

Building on this concept, the present work proposes a new approach to the utilization of industrial byproducts by repurposing high-value engineering material waste from different sectors: recycled polyamide 6 (rPA6) from automotive plastic washers, recycled graphite (rGra), and recycled CFRP (rCFRP) with an epoxy matrix from the aerospace industry. These materials were ground separately and compounded via twin-screw extrusion. The fillers (rGra and rCFRP) were incorporated individually and as hybrids (rCFRP:rGra, 1:1 by weight) at total contents of 5, 10, and 20 wt % into the rPA6 matrix. The aim was to develop multifunctional hybrid composites with excellent mechanical performance and promising properties for electrostatic discharge (ESD) control and electromagnetic compatibility (EMC) applications, particularly as microwave absorbing materials (MAMs).

## 2 Experimental Section

### 2.1 Materials and Processing

The materials used in this study were postindustrial scraps obtained from the aerospace and automotive sectors, as described below:

#### 2.1.1 Recycled Polyamide 6 (rPA6)

obtained from defective automotive plastic washers discarded due to dimensional issues, provided by a company located in São Bernardo do Campo, SP, Brazil. The washers were ground using a Rone S-200 knife mill equipped with a 3 mm sieve, producing fragments <3 mm, suitable for extrusion.

#### 2.1.2 Recycled Carbon Fiber-Reinforced Polymer (rCFRP)

aerospace scrap composed of 50.9 wt % T700SC 12K 660 carbon fiber and 49.1 wt % fast-curing epoxy resin (ER E732 FA/227, Toray), donated by a company located in São José dos Campos, SP, Brazil. The material, previously used in structural components, was mechanically ground to an average fiber length of 0.9 ± 0.5 mm, following the procedure detailed by dos Santos et al.^14^

#### 2.1.3 Recycled Graphite (rGra)

machining waste from aerospace brake components contaminated with cutting oil, supplied by an industry in São José dos Campos, SP, Brazil. The material was purified according to the method described by Zaggo et al.,^19^ and subsequently sieved to obtain particles between 212 and 425 mesh (33–212 μm).

The powders and fragments were dried in a vacuum oven at 80 °C for 24 h, then extruded using a corotating twin-screw extruder (AX Plásticos, AX16:40DR, *L*/*D* = 40) using a temperature profile of 220/235/245/245/255 °C, a screw rotation of 80 rpm, and a hopper feeding rate at 20 rpm. The extrudate was cooled to room temperature and pelletized. The pellets were subsequently dried again at 80 °C for 24 h under vacuum before compression molding and characterization.

Specimens for tensile testing (Type V – ASTM D638 ^21^) and rectangular samples (3 × 23 × 11 mm^3^) were prepared via compression molding in a hydropneumatic press (MH Equipamentos Ltd.a, PR8HP) at 260 °C under 7 bar for 5 min (1 min preheating without pressure, 3 min at 7 bar, 2 min cooling).

The composite formulations were designed following a factorial experimental design with total filler contents of 5, 10, and 20 wt %, including hybrids of rCFRP:rGra (1:1 by weight), maintaining the same total filler content as the single-filled composites (Table 1). A schematic representation of the processing methodology is shown in Figure 1.

**Figure 1:**
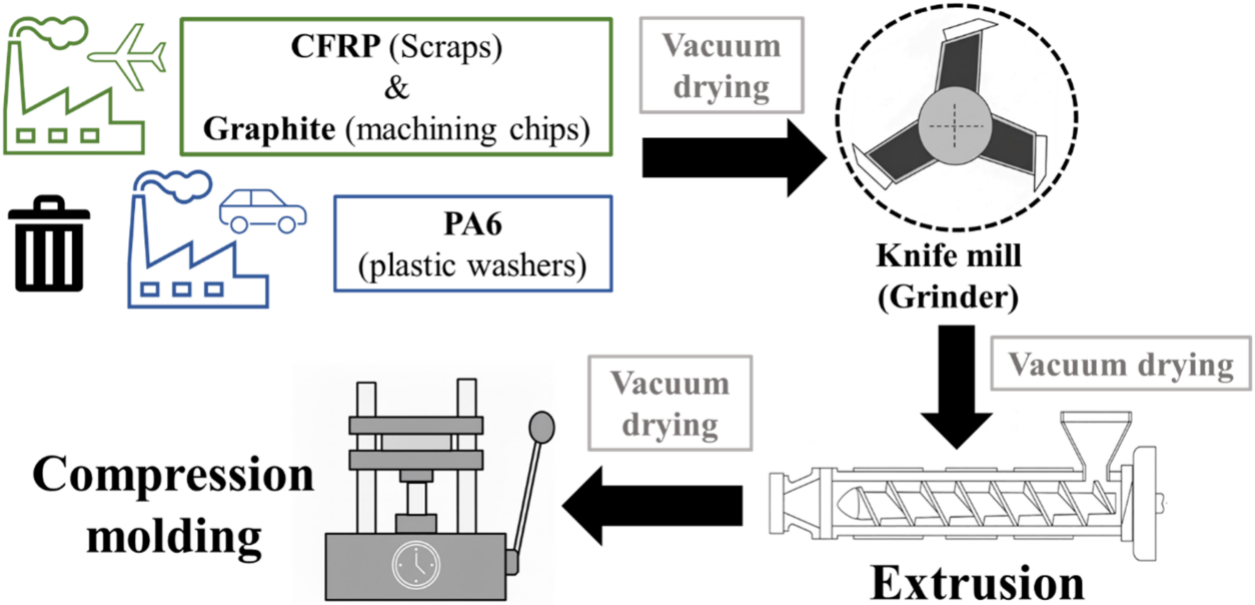
Methodology graphical scheme.

**Table 1:** Compositions and Nomenclature

Nomenclature	rPA6 (wt%)	rCFRP (wt%)	rGra (wt%)
rPA6	100		
rCFRP (5%)	95	5	
rCFRP (10%)	90	10	
rCFRP (20%)	80	20	
rGra (5%)	95		5
rGra (10%)	90		10
rGra (20%)	80		20
rCFRP/rGra (5%)	95	2.5	2.5
rCFRP/rGra (10%)	90	5	5
rCFRP/rGra (20%)	80	10	10

### 2.2 Characterizations

#### 2.2.1 Field-Emission Gun Scanning Electron Microscopy (FEG-SEM)

The morphology of rGra powder, rCFRP fragments, and cryo-fractured composite surfaces was analyzed using a TESCAN MIRA3 FEG-SEM operating at 5 kV. Samples were mounted on aluminum stubs with carbon tape and coated with a thin gold–palladium layer via sputtering to ensure conductivity.

#### 2.2.2 Base Materials Characterization

Structural characterization of rPA6, rGra, and rCFRP was performed using

##### 2.2.2.1 X-ray Diffraction (XRD)

Rigaku Ultima IV diffractometer with Cu Kα radiation (40 kV, 30 mA), using a nickel filter to remove Kβ radiation. Patterns were recorded over 2θ = 10–60° at a scanning speed of 10°/min.

##### 2.2.2.2 Raman Spectroscopy

HORIBA Lab-RAM HR Evolution with a 532 nm He–Ne laser, scanning 400–3000 cm^–1^.

##### 2.2.2.3 Fourier-Transform Infrared Spectroscopy (FT-IR)

PerkinElmer Frontier with universal attenuated total reflectance (UATR), scanning 400–4000 cm^–1^.

#### 2.2.3 Composites Characterization

##### 2.2.3.1 Rheology

Small-amplitude oscillatory shear (SAOS) tests were performed to evaluate the complex rheological behavior of rPA6 and its composites using a TA Instruments ARG2 rheometer with 25 mm parallel plates and a 1 mm gap, under nitrogen flow at 260 °C. Preliminary strain sweeps were performed to define the linear viscoelastic region, and a strain amplitude of 0.5% was selected for subsequent tests.

##### 2.2.3.2 Melt Flow Index (MFI)

The MFI was measured according to ASTM D1238 ^22^ at 245 °C under a 5 kg load, using a Hebert Lambert plastometer (model 4023).

##### 2.2.3.3 Differential Scanning Calorimetry (DSC)

The thermal behavior of postextrusion pellets was assessed on a TA Instruments Q2000 under nitrogen. Samples were sealed in aluminum pans and subjected to two heating cycles (10 – 270 °C at 10 °C/min) with an intermediate cooling. The first heating cycle was followed by an isothermal step at 270 °C for 3 min to erase the thermal history of the sample. From the obtained data the PA6 degree of crystallinity (*X*_c_) was calculated using the eq 1

1 $$X_{c}\left(\%\right)=\frac{{\Delta H}_{m}}{W.{\Delta H}_{m}^{o}}\times 100$$

Where *W* represents the weight fraction in the composition, Δ*H*_m_ represents the melting enthalpy calculate as the area of the melting DSC thermogram peak, and Δ*H*_m_^o^ represents the theoretical enthalpy for a 100% crystalline PA6 sample (190.8 J.g^–1^).^23^

##### 2.2.3.4 Tensile Testing

Five Type V specimens (ASTM D638–04 ^21^) were tested on an MTS Criterion 45 universal testing machine equipped with a 5 kN load cell, using a crosshead speed of 5 mm/min.

##### 2.2.3.5 Impedance Spectroscopy (IS)

AC conductivity was measured using a Solartron SI 1260 over 10–10^6^ Hz at 0.5 V. Tests were conducted on compressed films (0.1 mm) and bulk specimens (3.2 mm) to evaluate the electrical response.

##### 2.2.3.6 Electromagnetic Characterization

Shielding efficiency (SE), relative permittivity (ε_r_), relative permeability (μ_r_), and reflection loss (RL) were evaluated using a Vector Network Analyzer (VNA, Agilent PNA-L N5235A) in transmission mode with a W*R*-90 rectangular waveguide at X-band frequencies (8.2–12.4 GHz). Complex electromagnetic properties were calculated using the Nicolson-Ross-Weir (NRW) method following ASTM D5568–22a,^24^ after standard two-port calibration. SE values were derived from scattering parameters as described elsewhere.^25,26^ Additionally, one-port (reflectance-only) measurements were used to determine RL for nonoptimized sample thicknesses.

Using transmission line theory and the measured complex dielectric properties (ε_r_ and μ_r_), RL responses were extrapolated mathematically for various sample thicknesses based on the impedance matching theory (eqs 2 and 3).^26^

2 $$Z_{\mathrm{in}}=jZ_{0}\sqrt{\frac{\mu_{r}}{\varepsilon_{r}}}\tan h\left[\left(\frac{2\pi \mathrm{ft}}{c}\right)\sqrt{\mu_{r}\varepsilon_{r}}\right]$$

3 $$\mathrm{RL}\left(\mathrm{dB}\right)=20\log\left(\frac{Z_{\mathrm{in}}-Z_{0}}{Z_{\mathrm{in}}+Z_{0}}\right)$$

where: *j* stands for the imaginary number operator, *Z*_in_ intrinsic impedance of the material, *Z*_0_ the air impedance inside the transmission line set as 50 Ω during calibration, *t* is the materials layers thicknesses (which was extrapolated from 0.1 to 9.77 mm), *f* the waves frequency (8.2 to 12.4 GHz), and *c* the of light propagating in air (3.0 × 108 m/s).

## 3 Results and Discussion

### 3.1 Materials Characterization

The received materials were characterized after grinding to confirm their structural and chemical characteristics: rCFRP was analyzed by FEG-SEM and XRD, rGra by FEG-SEM, XRD, and Raman spectroscopy, and rPA6 by XRD, Raman spectroscopy, and FT-IR (Figure 2).

**Figure 2:**
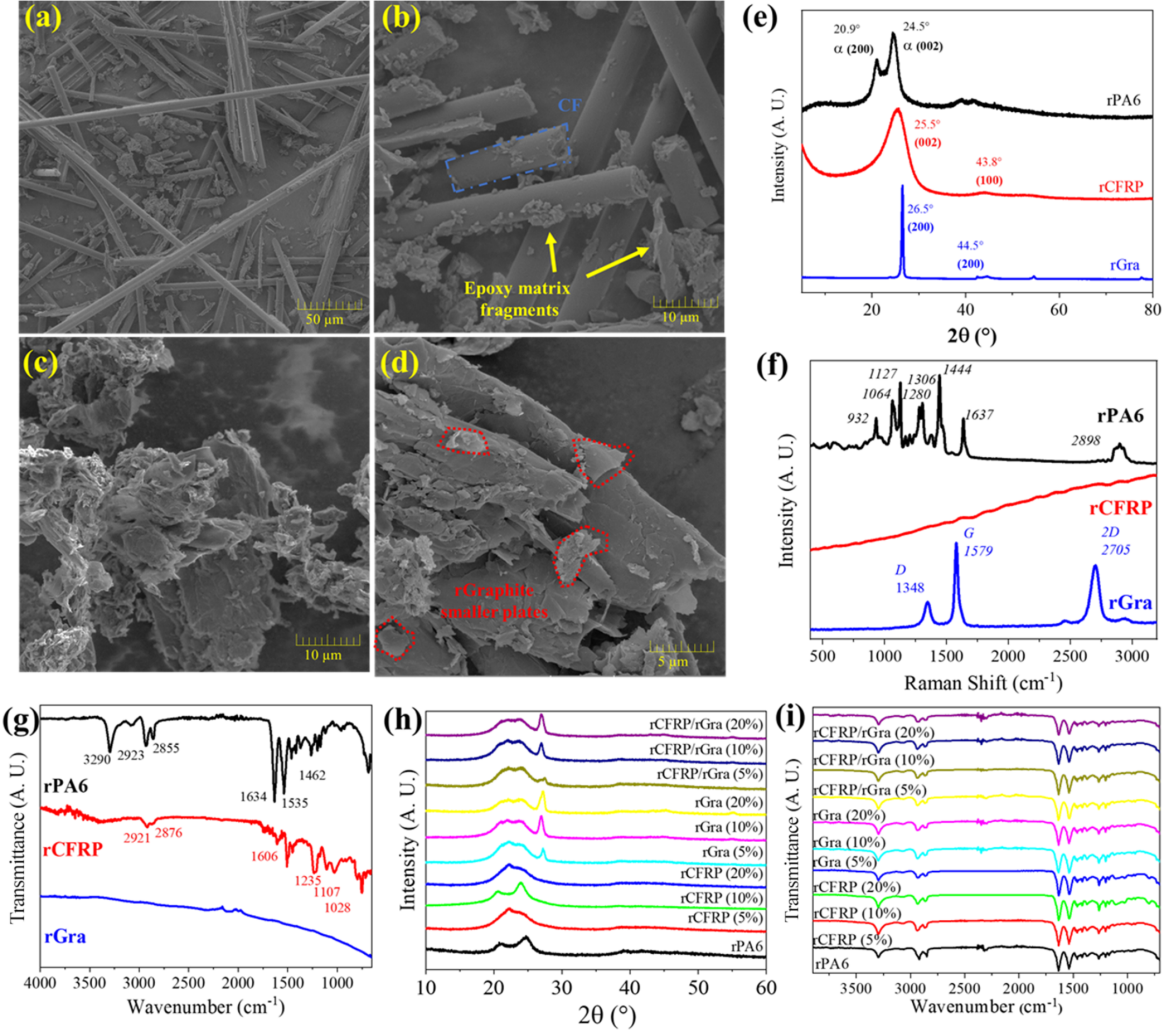
FEG-SEM morphology of (a, b) rCFRP and (c, d) rGra; (e) XRD, (f) Raman spectra and (g) FT-IR of the ground materials, (h) XRD diffractograms and (i) FTIR spectrum of the composites.

FEG-SEM images of rCFRP revealed fragments with different sizes of carbon fibers (CFs) and cured epoxy resin, appearing as large pieces or small resin residues on the fiber surfaces, with some regions still containing fibers bound together (Figure 2a–b). The rGra powder exhibited irregular and rough surfaces rather than smooth, graphitic-like microplates, with large flakes partially covered by fine debris. This morphology likely results from mechanical and/or thermal degradation during machining, milling, or purification steps (Figure 2c–d).

The XRD patterns of the ground materials exhibited the characteristic features of each component (Figure 2e). The rPA6 diffractogram showed two main peaks at 2θ = 20.9° and 24.5°, corresponding to reflections from the (200) and (002) crystalline planes of the α-polymorph of PA6,^12,27^ confirming that this crystalline phase is predominant in the recycled material. The rCFRP pattern presented a broad peak centered at 25.5°, assigned to the (002) plane of the sp^2^-hybridized carbon hexagonal lattice, typical of graphitic-like-oriented CFs. The peak broadness is attributed to the presence of cured epoxy resin and the random orientation of CFs within the composite residue. In contrast, the rGra displayed a sharp and intense diffraction peak near 26.5°, also associated with the (002) plane of sp^2^-hybridized carbon, but indicating a nonpreferentially oriented fine powder structure.^28^

Raman spectroscopy of rPA6 exhibited several characteristic vibrational bands of this polymer, enabling its differentiation from other polyamides such as PA6,6.^29^ The main bands were observed at 932 cm^–1^ and 1643 cm^–1^ (C–C=O stretching), 1064, 1127, and 2942 cm^–1^ (C–C backbone stretching), 1444 cm^–1^ (−CH_2_– bending), and 1280 cm^–1^ (C–N stretching and N–H bending). The rGra spectrum exhibited the typical fingerprint of graphite carbon materials,^30,31^ with three main bands: the D band at 1348 cm^–1^, associated with defects and sp^3^-hybridized carbon atoms near grain boundaries or surface functional groups; the G band at 1579 cm^–1^, the most intense, corresponding to the in-plane stretching of sp^2^-hybridized carbon atoms in a hexagonal lattice; and the 2D band at 2705 cm^–1^, related to interlayer interactions between graphene planes. The symmetric and sharp nature of the 2D band indicates a highly ordered graphite structure composed of large, well-stacked platelets.^30^

The rCFRP absorbed the Raman laser energy too strongly over the entire spectral range, rendering the Raman spectra unsuitable for reliable analysis under the applied conditions. This behavior is attributed to the highly ordered sp^2^-hybridized carbon structure of the carbon fibers, consisting of parallel hexagonal lattices that are continuous along the fiber length and highly conductive, leading to efficient energy absorption. A proper evaluation would require different analytical parameters, which would preclude direct comparison with the other samples; therefore, such analysis was considered beyond the scope of this work.

The FT-IR spectrum of rPA6 exhibited the characteristic absorption bands of the polyamide family,^32^ particularly those associated with amide groups: 3290 cm^–1^ (N–H stretching), 1535 cm^–1^ (N–H deformation and C–N stretching), and 1634 cm^–1^ (C=O stretching of amide I). The bands at 2923 and 2855 cm^–1^ correspond to – CH_2_– stretching vibrations of the polymer backbone. Although FT-IR is a powerful technique for polymer identification, distinguishing between different polyamides based solely on their spectra remains challenging. Nevertheless, the presence of a band around 1462 cm^–1^ supports the identification of PA6.^32^ Taken together, the XRD, Raman, and FT-IR analyses consistently confirm that the recycled polymer corresponds to polyamide 6 (rPA6), a conclusion further corroborated by its characteristic DSC thermal transitions, discussed in the following section. The FT-IR spectrum of the rCFRP exhibited characteristic absorption bands of cured epoxy resin,^33,34^ confirming the presence of the matrix. These included bands at 1107 and 1606 cm^–1^, associated with aromatic C=C vibrations; 1235 cm^–1^ related to C–O stretching; 1028 cm^–1^ attributed to ether (−O−) linkages; and bands at 2921 and 1507 cm^–1^ corresponding to C–H vibrations.^33,34^ In contrast, the rGra spectrum appeared essentially as a baseline, as this material strongly absorbs in the infrared region, preventing reliable FT-IR characterization under the applied conditions. A similar, though less pronounced, absorption behavior was also observed for the carbon fibers, which attenuated some bands in the rCFRP spectrum.

Overall, the structural and chemical characterizations verify the correct identification of the recovered materials and indicate no significant molecular degradation during the grinding or preparation processes. The most intense characteristic XRD peaks and FTIR bands of the base materials were also observed in the composites (Figure 2h–i), corroborating their compositions. No significant peak or band shifts were detected, and the intensity variations were primarily associated with the relative content of each component in the composites.

### 3.2 Recycled Composites: Morphologies, Rheological, and Thermal behavior

The rPA6 (Figure 3a,e) exhibited a typical thermoplastic cryofracture morphology, characterized by a relatively smooth fracture with some spherical domains visible at higher magnification (Figure 3e), possibly related to minor additives or trace polymeric impurities. Their low content suggests negligible influence on the overall behavior. The rCFRP (20%) (Figure 3b,f) morphology revealed well-dispersed carbon fibers within the rPA6 matrix and occasional fiber pull-outs (highlighted with yellow arrows). At higher magnification (Figure 3f), the fibers appeared well-wetted by PA6, with polymer coating regions of cured epoxy resin (ER), evidence of strong interfacial adhesion between the recycled fibers and the thermoplastic matrix. Both morphologies were similar to those observed by Dong et al.^9^ for injection-molded CF/PA6 composites, reinforcing the efficient interfacial interaction achieved in the present recycled system.

**Figure 3:**
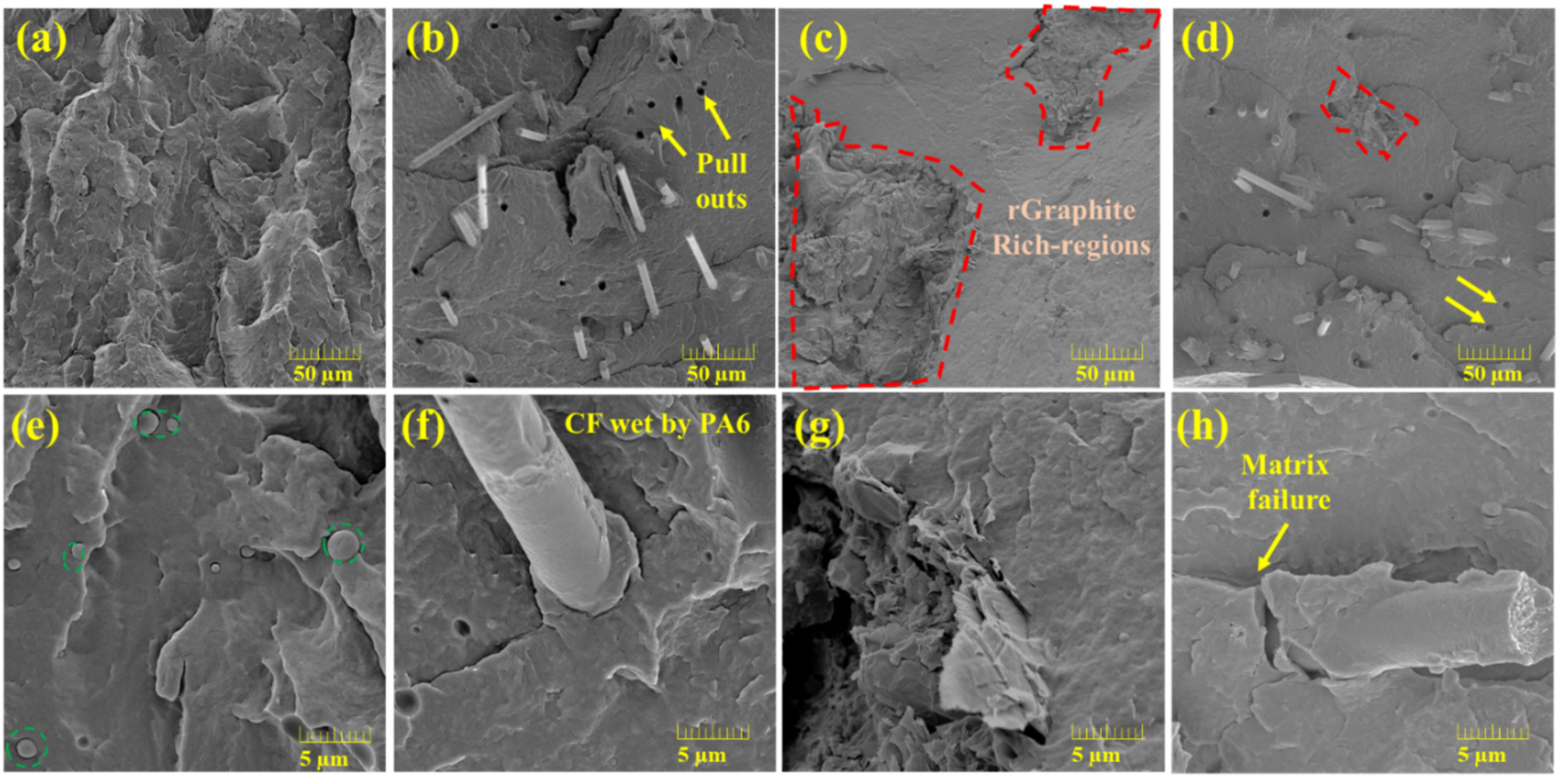
FEG-SEM morphology of the rPA6 and the composites: (a) and (e) rPA6; (b) and (f) rCFRP (20%); (c) and (g) rGra (20%); (d) and (h) rCFRP/rGra (20%).

In contrast, the rGra (20%) (Figure 3c,g) composite exhibited graphite-rich regions (highlighted in red dashed lines), indicating limited filler dispersion through the matrix. High-magnification images showed voids and dry interfacial zones between the graphite platelets and the polymer matrix, further confirming the poor wetting of this filler with rPA6.

For the hybrid rCFRP/rGra (20%) composite (Figure 3d,h), the CF were uniformly distributed without preferential orientation, while graphite agglomerates appeared smaller and more finely dispersed, suggesting that combining both fillers enhanced the overall dispersion state. At higher magnification (Figure 3h), the fibers were again well wetted by the polymer, and only minor matrix microcracks were observed, possibly originating from localized stress concentration during cryofracture.

The rheological behavior in the SAOS regime reveals important correlations between filler addition and polymer–filler interactions, reflecting the morphological features observed.^35^ Unlike nanocomposites, where high surface area typically induces pronounced changes in the rheological response, the micrometer- to millimeter-sized fillers used in this study caused only modest variations in complex viscosity (η)* and complex modulus (*G*)* at 5 and 10 wt % (Figure 4a–c).

**Figure 4:**
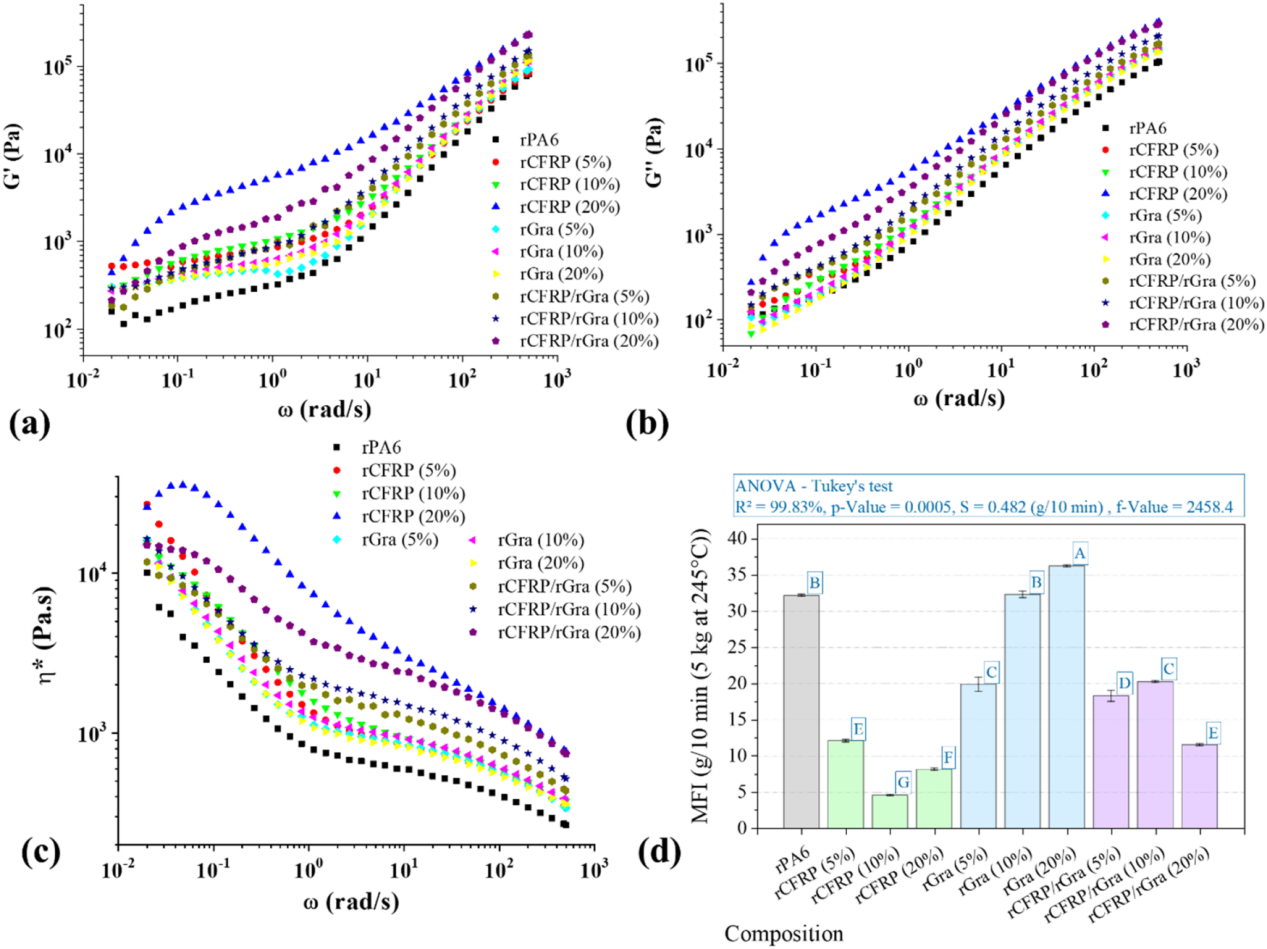
Rheological characterization of the composites. SAOS: complex shear modulus (G*) **((a)** Storage (*G*′), (b) Loss (*G*″)); (c) Complex viscosity (η*), and (d) Melt flow index (MFI).

At higher filler content (20 wt %) and low angular frequencies (ω), distinct behaviors were observed. The rCFRP-filled composite significantly increases η* and G*, reflecting restricted chain mobility of rPA6 matrix caused by the fiber aspect ratio, the presence of cured epoxy, and partial interfacial interactions between the matrix and the recycled fibers. In contrast, the rGra-filled composite showed minimal impact even at 20 wt %, consistent with the intrinsic lubricating character, low polarity, and plate-like morphology of graphite, which reduce its interactions with the PA6 matrix.

The hybrid rCFRP/rGra composites exhibit an intermediate rheological response: η* and G* were lower than those of the 20 wt % rCFRP but significantly higher than those of the 10 wt % rCFRP. This may result from π–π interactions between sp^2^-hybridized carbon atoms of rGra and the exposed carbon fiber surfaces, improve dispersion within the polymer matrix, and reduce the lubricating effect of rGra. Similar behavior was observed for other carbon-based materials when added together in hybrid composites.^26^

The melt flow index (MFI) results further support these observations (Figure 4d). The incorporation of rCFRP decreases MFI due to restricted molecular mobility, while rGra slightly increases it, reflecting the formation of low-friction regions and limited interfacial adhesion between rPA6 and rGra. Hybrids with 5 and 10 wt % of total filler exhibited higher MFI values than their rCFRP-only composites, indicating a promising balance between processability and reinforcement due to the combined effects of both fillers.

DSC results are presented in Figure 5 and summarized in Table 2. PA6 is a semicrystalline engineering thermoplastic with high mechanical strength, a glass transition temperature (*T*_g_) of ∼50–60 °C, and a melting temperature (*T*_m_) around 220 °C, primarily attributed to the strong intermolecular hydrogen-bonding between amide and carboxyl groups.^12^ PA6 also exhibits polymorphism, with the α-phase (*T*_m_ ∼ 220–224 °C) being the most thermodynamically stable form, and the γ-phase (*T*_m_ ∼ 215–220 °C) typically promoted under constrained crystallization conditions.^27,28^

**Figure 5:**
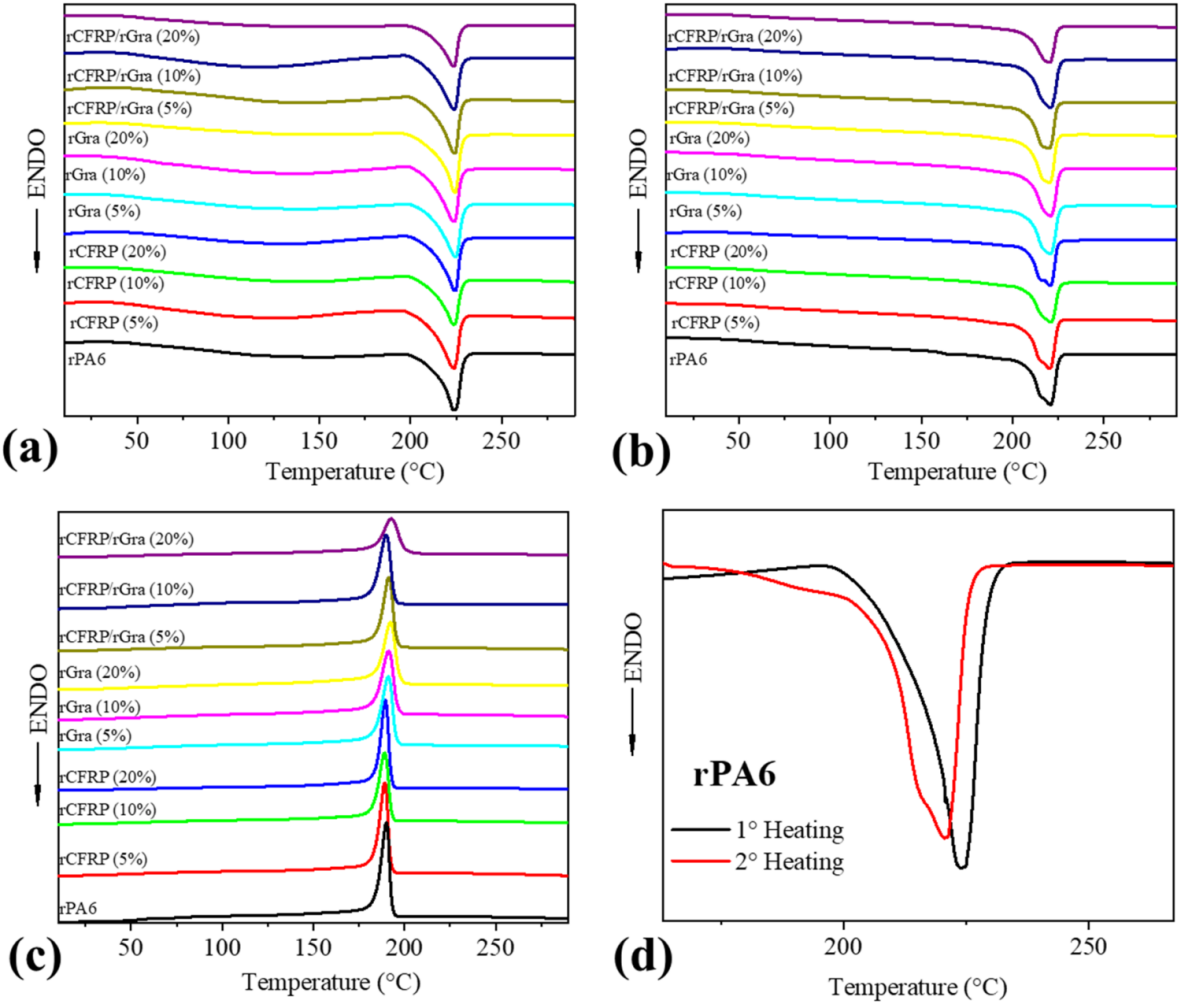
DSC thermograms (a) 1° heating, (b) 2° heating, (c) cooling, and (d) a 1° and 2° heating of rPA6 superposed to highlight the differences.

**Table 2:** DSC Analysis Results

	1° heating				cooling		2° heating			
compositions	*T*_g_ (°C)	*T*_m_ (°C)	Δ*H*_m_ (J/g)	*X*_c_ (%)	*T*_c_ (°C)	Δ*H*_c_ (J/g)	*T*_g_ (°C)	*T*_m_ (°C)	Δ*H*_m_ (J/g)	*X*_c_ (%)
rPA6	49	224	55	29	190	62	53	221	58	30
rCFRP (5%)	54	224	57	31	189	60	58	220	58	32
rCFRP (10%)	52	224	45	26	189	49	56	221	48	28
rCFRP (20%)	56	224	50	33	189	55	57	221	49	32
rGra (5%)	49	224	51	28	191	56	53	221	55	31
rGra (10%)	58	224	50	29	191	57	56	221	56	33
rGra (20%)	53	224	55	36	192	56	55	220	53	35
rCFRP/rGra (5%)	55	224	51	28	191	54	58	220	51	28
rCFRP/rGra (10%)	53	224	51	30	190	57	58	221	55	32
rCFRP/rGra (20%)	53	224	37	25	193	40	56	220	41	27

For rPA6, DSC analyses revealed *T*_g_ = 49 °C and *T*_m_ = 224 °C, with a degree of crystallinity (*X*_c_) ∼ 29% during the first heating cycle. In the second heating, a slight increase in *T*_g_ and a decrease in *T*_m_ were observed, likely associated with an increased proportion of the γ-phase, induced by the relatively slow cooling rate (10 °C/min) of the DSC experiment. This phenomenon, evidenced by the appearance of a shoulder in the melting endotherm (Figure 4d), is mainly of scientific interest, since conventional melt-processing conditions do not reproduce such low cooling rates.

For the recycled composites, no significant thermal changes were observed compared to rPA6. Slight increases of 1–3 °C in *T*_g_ (second heating) and crystallization temperature (*T*_c_) were noted for rGra-containing composites. Additionally, rGra (20 wt %) exhibited a minor nucleating effect, increasing *X*_c_ by ∼5%. Overall, all composites followed the same trend of reduced *T*_m_ in the second heating, confirming the cooling-rate influence on γ-phase formation, but these changes are not practically significant.

### 3.3 Mechanical Behavior of the Recycled Composites

The mechanical properties of the composites are shown in Figure 6a–b and summarized in Table 3 with statistical evaluation. As expected, rPA6 exhibited high mechanical strength due to strong secondary interactions, with an elastic modulus (*E*) of 1.8 GPa and an ultimate tensile strength (UTS) of 59 MPa, significantly higher than typical polyolefins,^23^ as an example.

**Figure 6:**
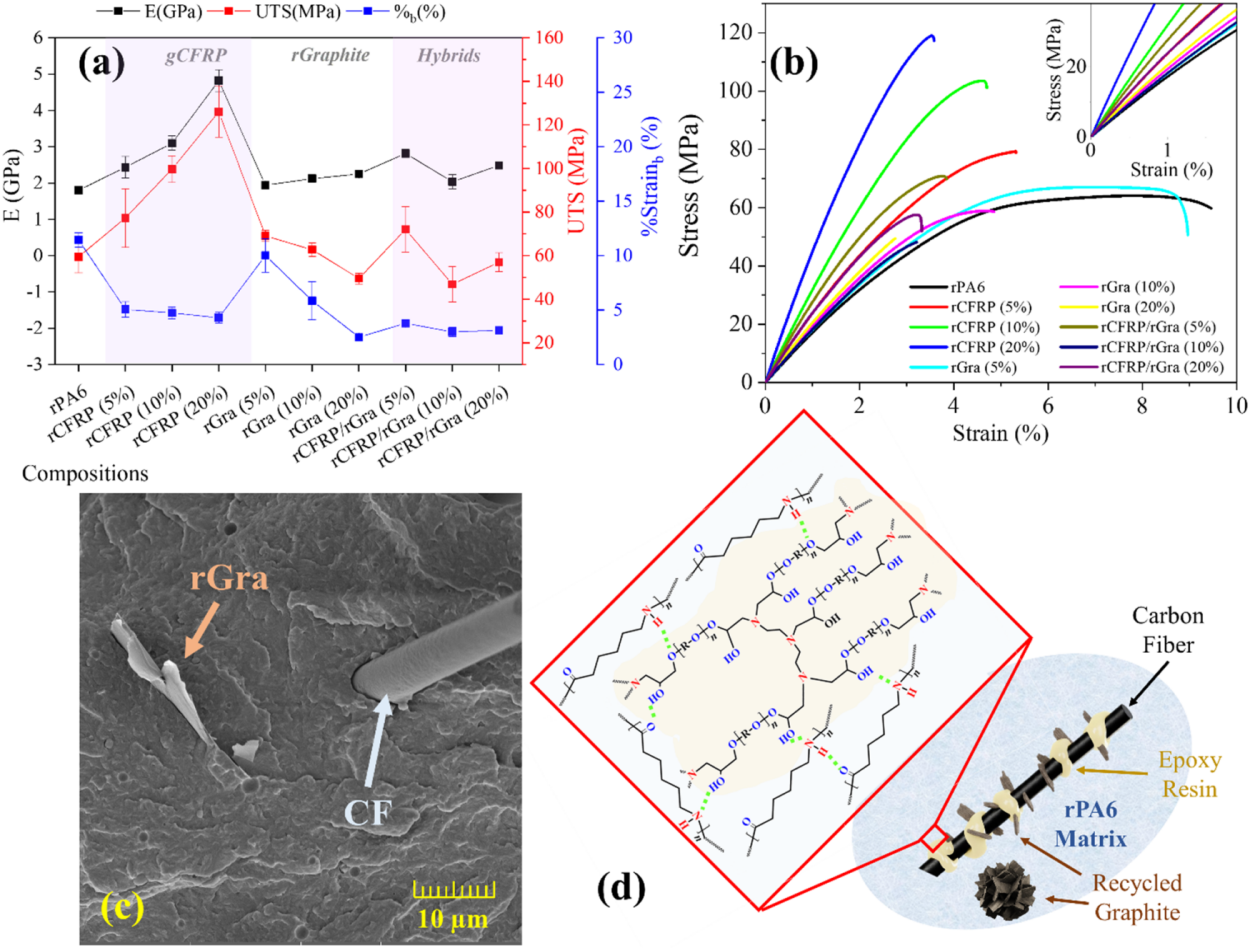
Tensile strength test results: (a) Mechanical properties summary figure, (b) stress–strain curves (one sample representing each composition); (c) FEG-SEM morphology of rCFRP/rGra (20%) representing low adhesion of CF at the PA6 in an area without epoxy covering and a rGra plate nearby, and (d) a schematic representation of the morphology indicating possible hydrogen-bonds between rPA6, and cured epoxy resin (ER).

**Table 3:** Mechanical Properties of the Composites: Elastic Modulus (*E*), Ultimate Tensile Strength (UTS), and Strain at Break (%b)^a^

compositions	*E* (GPa)	UTS (MPa)	%b (%)
rPA6	1.80 ± 0.11^G^	59 ± 7^CDE^	11.44 ± 0.67^A^
rCFRP (5%)	2.44 ± 0.3^CDE^	77 ± 13^C^	5.08 ± 0.72^BC^
rCFRP (10%)	3.10 ± 0.2^B^	100 ± 6^B^	4.74 ± 0.54^BCD^
rCFRP (20%)	4.82 ± 0.3^A^	126 ± 12^A^	4.30 ± 0.51^BCDE^
rGra (5%)	1.94 ± 0.08^FG^	69 ± 3^CD^	10.01 ± 1.53^A^
rGra (10%)	2.12 ± 0.08^DEFG^	63 ± 3^CDE^	5.86 ± 1.73^B^
rGra (20%)	2.25 ± 0.09^DEF^	50 ± 2^E^	2.51 ± 0.19^E^
rCFRP/rGra (5%)	2.82 ± 0.11^BC^	72 ± 10^CD^	3.79 ± 0.21 B^CDE^
rCFRP/rGra (10%)	2.04 ± 0.19^EFG^	47 ± 8^E^	3.00 ± 0.43^DE^
rCFRP/rGra (20%)	2.49 ± 0.06^CD^	57 ± 4^DE^	3.14 ± 0.04^CDE^
ANOVA-Tukey tests info (α = 0.05–bilateral)			
*E*: *p*-Value = 0.0006, *F*-Value = 101.97, *S* = 0.174 GPa, and *R* ^2^ = 96.83%			
UTS: *p*-Value = 0.0009, *F*-Value = 38.15, *S* = 7.8 MPa, and *R* ^2^ = 91.96%			
%b: *p*-Value = 0.0008, *F*-Value = 50.84, *S* = 0.844%, and *R* ^2^ = 93.85%			

a Average values ± standard deviation with the post-hoc Tukey’s test groups indicated by superscript letters.

The addition of rCFRP was the main contributor to improved mechanical performance. Both *E* and UTS increased almost linearly with rCFRP content, reaching *E* = 4.8 GPa and UTS = 126 MPa at 20 wt % rCFRP, corresponding to gains of 168% in elastic modulus and 114% in strength relative to rPA6. This remarkable effect of CF increasing PA6 mechanical properties has been widely reported in the literature.^6,9–11^ However, the novelty of the present system lies in the fact that such enhancement was achieved using short, discontinuous recycled fibers, likely facilitated by secondary hydrogen bonding between rPA6 and the cured epoxy phase within the rCFRP particles (Figure 6d), indicating effective interfacial adhesion despite the heterogeneous filler morphology.

In contrast, rGra addition did not significantly affect E or UTS but reduced strain at break (%b) linearly. At 5 wt % rGra, the strain remained similar to rPA6, preserving ductility. Poor matrix–filler affinity led to large agglomerates, which limited stress transfer and, at higher contents, could act as microcracks, further reducing ductility.

For the hybrid rCFRP/rGra composites, two main factors influenced the mechanical behavior: the lower effective CF content (2.5–10 wt %), which plays the main role in improving mechanical properties; and interactions at exposed fiber areas without cured epoxy, which can weaken adhesion relative to rCFRP-only composites and interact with rGra via interactions of π–π type (exemplified schematically in Figure 6d). Morphological analysis confirmed the presence of voids and interfacial gaps between rGra and the matrix in some hybrid samples (Figure 6c). Among hybrids, only the rCFRP/rGra (5%) composite (2.5 wt % of each filler) showed promising mechanical performance, comparable to rCFRP (5%) in both *E* and UTS.

### 3.4 Recycled Composites: Electric Conductivity and Electromagnetic Behavior

Impedance spectroscopy (IS) was performed on compression-molded films (∼0.2 mm thick) to evaluate electrical conductivity for antielectrostatic discharge (ESD) applications.^19^ Both rCFRP and rGra exhibited intrinsic electrical conductivity arising from their sp^2^-hybridized carbon hexagonal lattice, whereas the PA6 matrix shows highly insulating behavior associated with its covalently bonded polymer backbone, which lacks mobile charge carriers. When incorporated into the polymer matrix, these fillers can establish continuous conductive networks once their concentration surpasses the percolation threshold, enabling efficient charge dissipation.^36^

As shown in Figure 7a, the incorporation of rCFRP significantly increased the electrical conductivity, even at low filler contents, reaching 10^–5^–10^–6^ S.cm^–1^, likely due to the long fiber fragments where only a few in contact can percolate the film thickness. In contrast, rGra-filled composites remained nearly insulating at 5 and 10 wt %, while 20 wt % exhibited semiconductive behavior (∼2 × 10^–3^ S.cm^–1^), indicating that the percolation threshold is reached at higher loadings due to the smaller particle size and reduced aspect ratio than the CF in the rCFRP fragments. This behavior of rGra is consistent with the findings of *Zaggo et al*.,^19^ who also reported that the use of compatibilizer agents to improve filler dispersion can reduce the rGra content required to achieve enhanced electrical conductivity.

**Figure 7:**
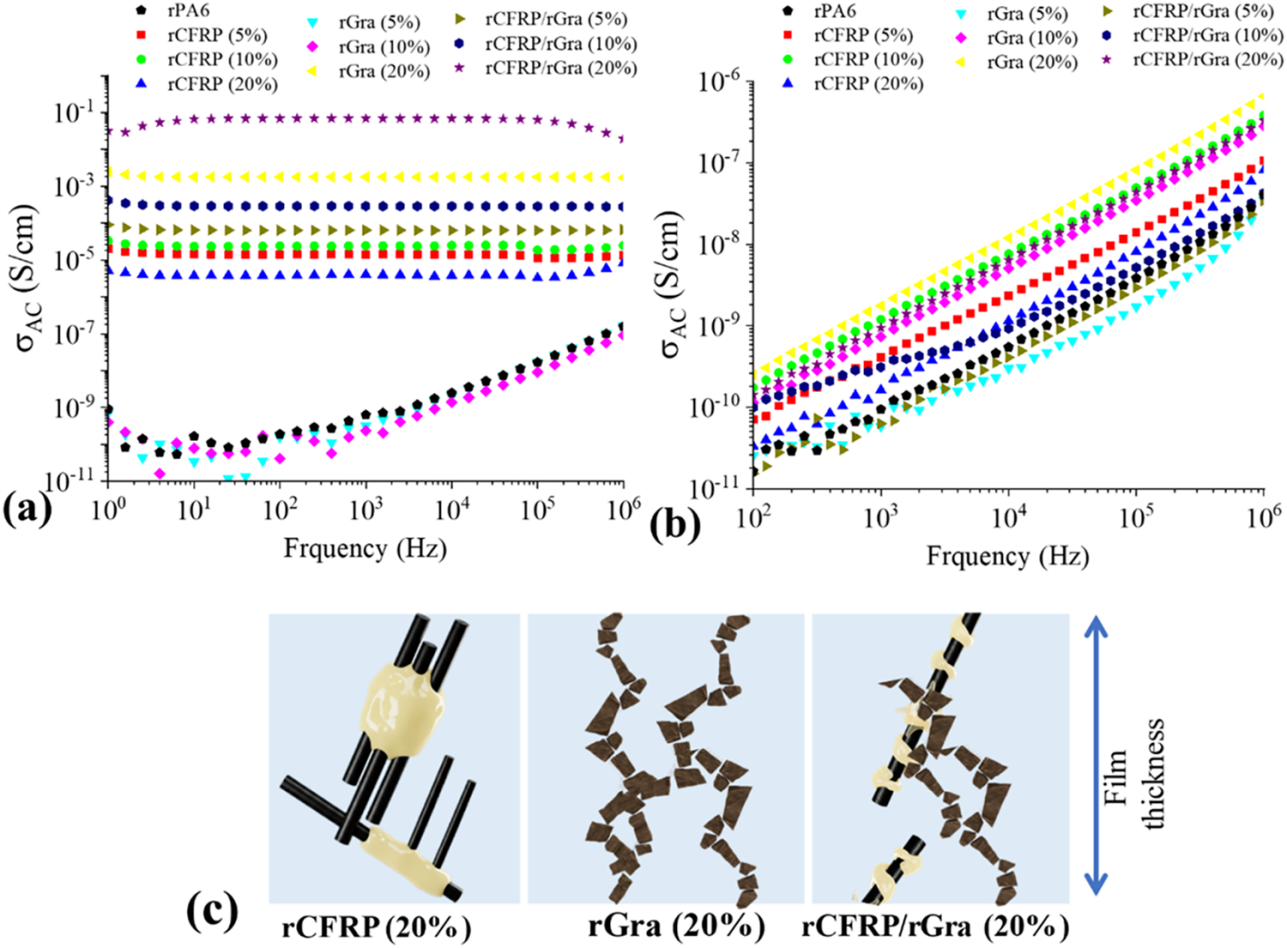
AC Electrical conductivity of: (a) Films and (b) bulk samples; (c) schematic representation of the network formation through film thickness.

The hybrid rCFRP/rGra composites (5 and 10 wt %) showed slightly higher conductivity than rCFRP-only composites, while the 20 wt % hybrid exhibited the highest conductivity among all formulations, indicating a synergistic effect between the fibrous and platelet fillers in promoting the formation of three-dimensional conductive networks.

For the bulk specimens, most composites remained insulating, with slight increases in conductivity at higher filler contents (Figure 7b). The anisotropy and conductivity differences observed between films and bulk samples are consistent with previous reports for polymer carbon-based composites.^37^ These effects are typically attributed to processing effects, such as the formation of an insulating surface layer during compression, or the influence of agglomerates, which impede continuous pathways in thicker samples. In thin films, the thickness is comparable to the size of rGra particles or rCFRP fragments, facilitating short conductive pathways (Figure 7c).

The EMI shielding effectiveness (SE) of the composites exhibited a strong dependence on both filler content and filler type^25^ (Figure 8a–c). For the rCFRP-based composites, the reflection component (SE_R_) increased from ∼2 dB (rPA6) to 4–5 dB at low filler contents, while higher contents led to a frequency-dependent decrease in SE_R_, particularly at 20 wt %. In contrast, the absorption component (SE_A_) gradually increased with filler content, with rCFRP (20 wt %) showing the highest overall attenuation (SE_T_ = 7.5–10.7 dB) across the evaluated frequency range.

**Figure 8:**
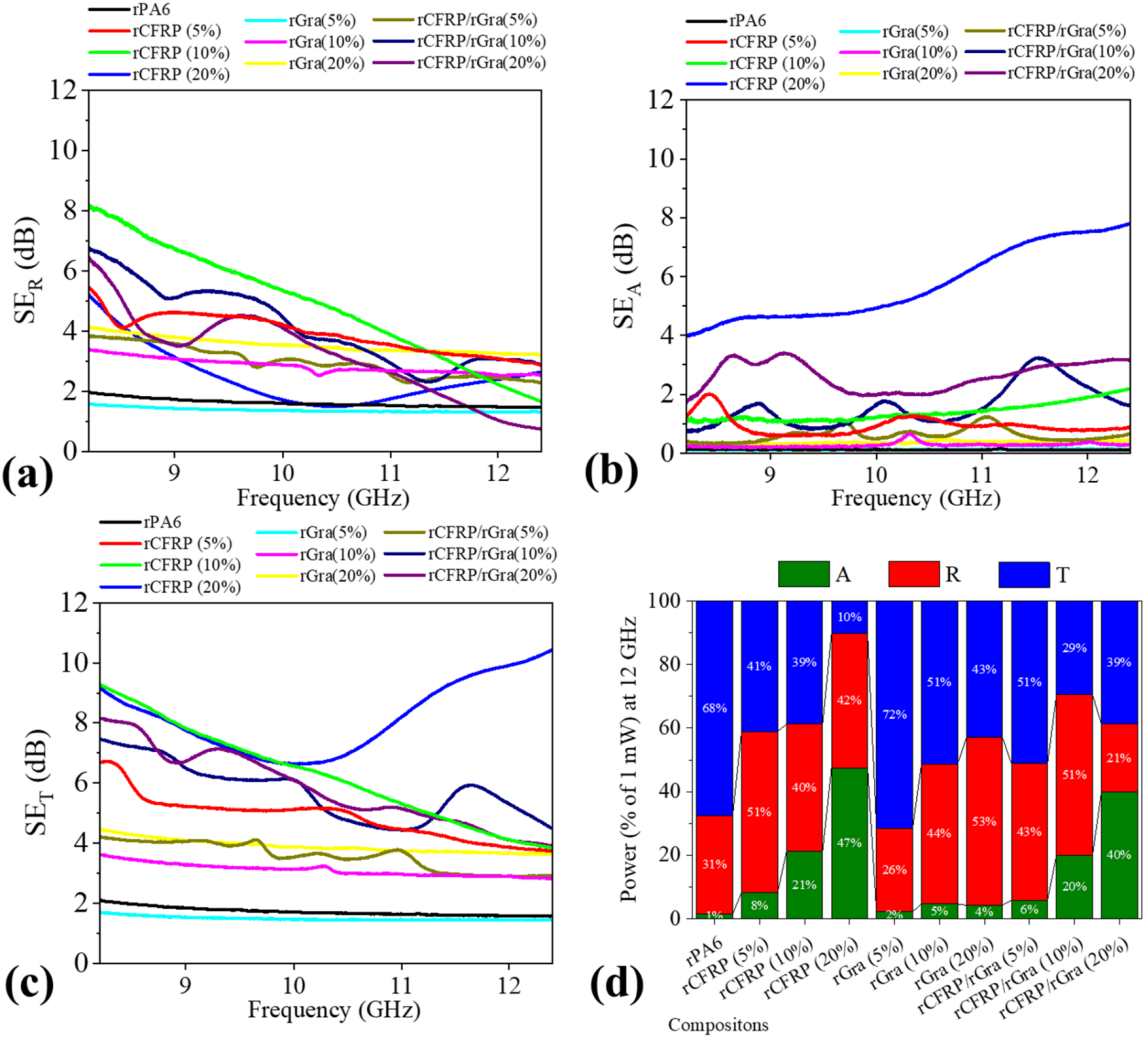
Electromagnetic interference shielding response performance: (a) Attenuation by reflection component (SE_R_), (b) attenuation by absorption component (SE_A_), and (c) total attenuation component (SE_T_); (d) the power coefficients distribution at 12 GHz, showing absorbance (A), reflectance (R), and transmittance (T).

The SE_R_ is mainly governed by the surface impedance mismatch between the composite and air, which is linked to electrical conductivity. In contrast, SE_A_ arises from internal losses, such as conduction and interfacial polarization between conductive fragments or agglomerates.^38^ The morphology and fragment-size distribution of rCFRP influence these mechanisms. The average fiber length and aspect ratio of the CF are crucial parameters for the frequency-dependent SE behavior of discontinuous CF-based composites, where longer fibers are more likely to promote higher SE response.^36^ This behavior may explain the low SE values obtained, since these fibers had an average length of 0.9 mm, which is considerably short.

Composites with rGra exhibited lower but more stable EMI attenuation. The 5 wt % composition behaved similarly to rPA6, while 10 and 20 wt % produced modest increases in SE_T_ to 3.8 and 4.1 dB, respectively, mostly due to reflection. Hybrid rCFRP/rGra composites displayed intermediate and more oscillatory SE_T_ profiles, likely resulting from their heterogeneous morphology and combined network of fillers with distinct shapes and conductivities. The SE_A_ component increased with filler content, consistent with the rCFRP trend.

At 12 GHz, the power coefficient distribution (A, R, T) (Figure 8d) followed the same tendencies. The lowest transmission (*T* ≈ 10%) was obtained for rCFRP (20 wt %), still below the 20 dB commercial benchmark (<1% transmission).^25^ Comparable attenuation was reported by Pu et al.,^7^ who achieved ∼5 dB for 20 wt % recycled CFRP in epoxy, requiring an additional 5 wt % MWCNT to reach commercial performance levels. Similarly, increasing filler content or incorporating conductive nanofillers could further enhance EMI shielding in these recycled composites.

Figure 9 presents the complex electromagnetic parameters (ε_r_ and μ_r_) of the composites. The results corroborate the previously discussed attenuation mechanisms: an increase in ε_r_ is associated with higher total shielding effectiveness (SE_T_). In contrast, a higher dielectric loss tangent (ε″/ε′) corresponds to samples exhibiting greater absorption (SE_A_) components. For all compositions, μ_r_ remained close to unity across the entire frequency range, confirming the nonmagnetic nature of these systems and indicating that electromagnetic attenuation arises predominantly from conductive rather than magnetic losses, consistent with the presence of sp^2^ carbon–based fillers.

**Figure 9:**
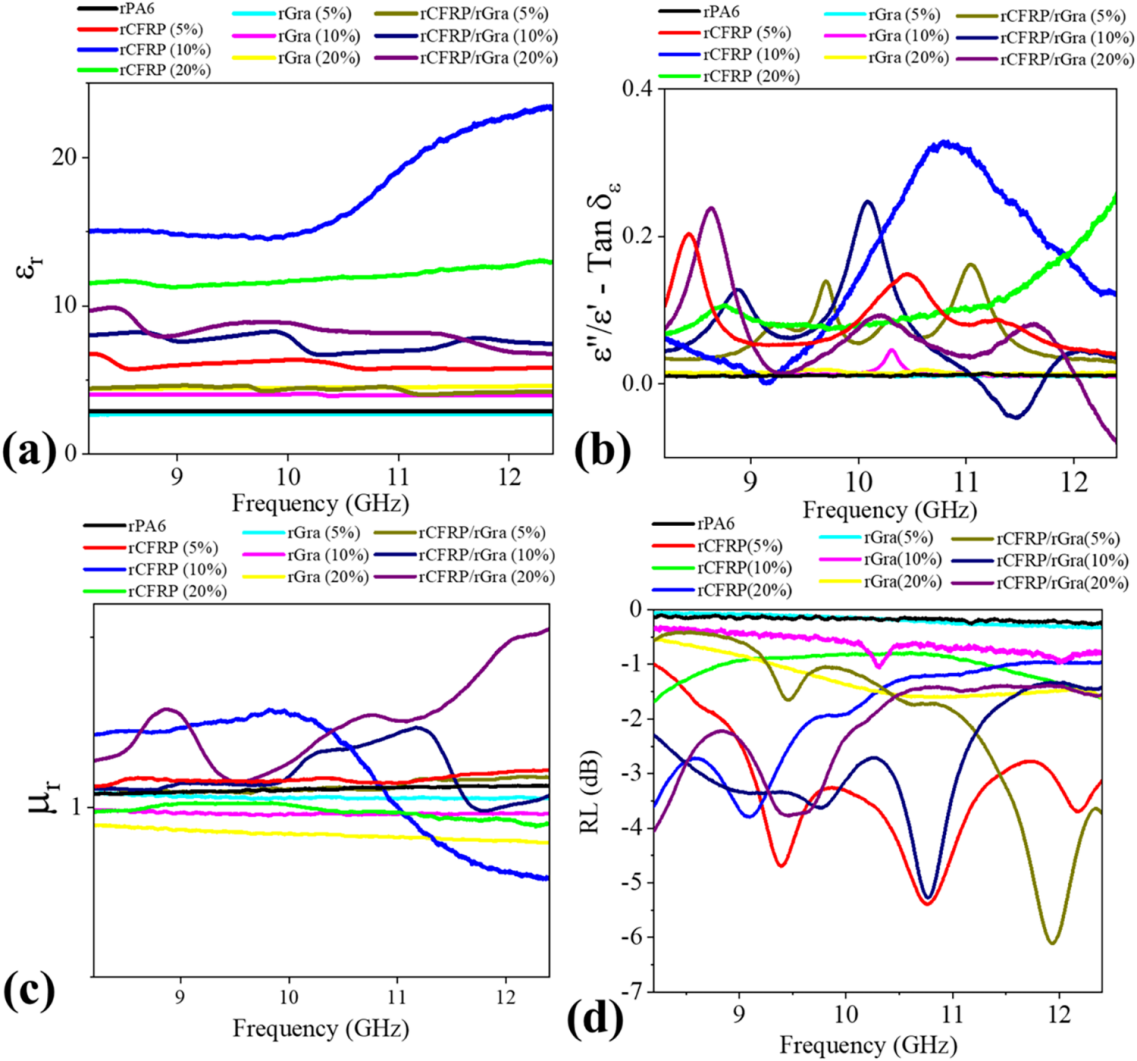
Complex electromagnetic properties obtained by NRW (a) relative permittivity (ε_r_), (b) loss tangent (ε″/ε′), (c) relative permeability (μ_r_), and (d) measured Reflection Loss (RL) for the samples.

Another important aspect of electromagnetic functionality is the reflection loss (RL) response, which is particularly relevant for microwave absorber materials (MAMs).^39,40^ These materials are typically engineered to operate in combination with highly conductive layers to suppress back-reflection toward the source. The RL responses of the composites are shown in Figure 9d. As observed, RL behavior is more complex than the shielding response, depending not only on filler content but also on the dielectric loss ratio (ε″/ε′). Interestingly, some samples exhibited multiple absorption bands, which may be attributed to the presence of filler particles of different sizes within the composite.

Although none of the measured samples achieved the practical absorption criterion of RL < −10 dB when evaluated as monolithic samples, this limitation is primarily attributed to the nonoptimized sample thicknesses used in the experimental setup. To further explore their intrinsic absorption potential, electromagnetic simulations were carried out based on the measured complex permittivity and permeability data. These simulations aimed to identify whether any of the compositions could achieve effective absorption bands (RL < −10 dB) within the X-band frequency range under optimized thickness conditions (Figure 10).

**Figure 10:**
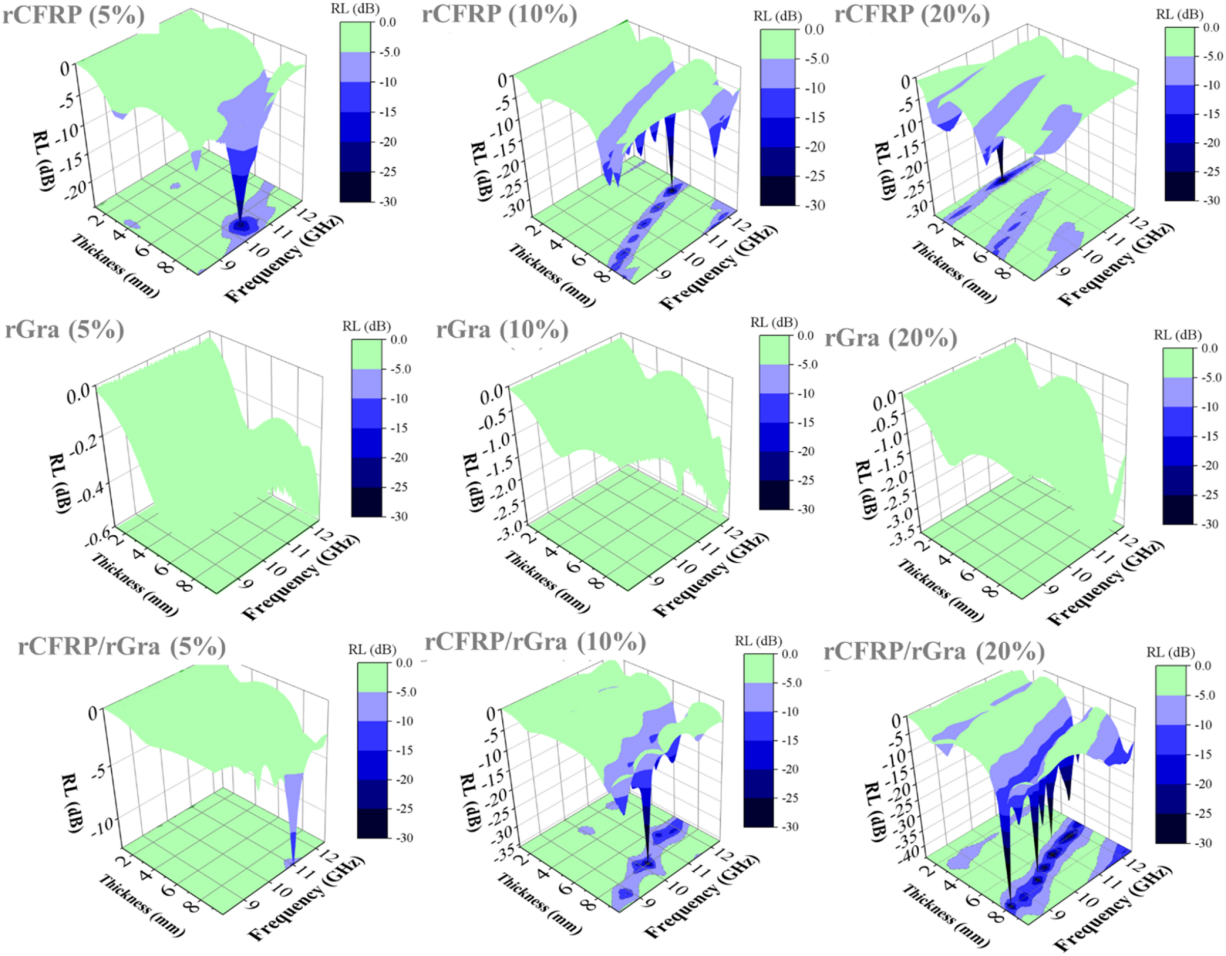
Reflection Loss behavior simulations for all composites.

According to the simulated reflection loss profiles at different sample thicknesses, all composites containing rCFRP at all concentrations, as well as the hybrid formulations with 10 and 20 wt % total filler content, exhibited promising absorption regions suitable for potential application as monolithic microwave absorber materials (MAMs) with Dallenbach-type topology. These regions, represented by the medium-blue to black areas in Figure 10, correspond to frequencies where RL values approach or exceed the desired absorption threshold. It is worth noting, however, that the optimized sample thicknesses required to achieve these responses may not be feasible for all practical MAM applications due to weight or design constraints.

## 4 Conclusions

Recycled PA6-based composites reinforced with recycled carbon fiber reinforced polymer (rCFRP) and recycled graphite (rGra) were successfully developed and characterized, demonstrating that both waste-derived fillers can impart valuable multifunctional properties to the polymer matrix. Rheological analysis revealed a slight increase in complex viscosity and a marked reduction in MFI upon rCFRP addition, while rGra at higher contents (10 and 20 wt %) maintained values close to those of neat rPA6. The addition of rCFRP resulted in a remarkable mechanical enhancement, with an increase of up to 168% in elastic modulus and 114% in ultimate tensile strength compared to rPA6, attributed to the strong interfacial affinity between polymer and the cured epoxy fragments. In contrast, rGra additions produced negligible reinforcement but preserved ductility at low contents, with limited interfacial bonding leading to reduced stress transfer at higher loadings. Electrical and electromagnetic analyses confirmed that rCFRP promoted conductivity even at low contents due to its elongated morphology, whereas rGra required higher loadings (>10 wt %) to achieve the percolation threshold. Hybrid rCFRP/rGra composites exhibited intermediate behavior, showing partial synergism between fillers. Electromagnetic interference (EMI) shielding analysis revealed total shielding effectiveness (SE_T_) reaching ∼10.7 dB for rCFRP (20 wt %). The composites remained nonmagnetic (μr ≈ 1), and the dielectric loss (ε″/ε′) correlated with higher absorption performance. Reflection loss (RL) simulations indicated that composites with rCFRP, particularly at higher loadings or in hybrid formulations, can exhibit absorption regions suitable for monolithic microwave absorber materials (MAM) applications when optimized thicknesses are used. Overall, this study provides strong scientific and technical evidence for the development of high-value, multifunctional composites from postindustrial waste sources, combining strong mechanical performance with promising electrical conductivity and electromagnetic functionality, contributing to circular materials engineering and sustainable manufacturing.
